# Nitrative Stress and Auditory Dysfunction

**DOI:** 10.3390/ph15060649

**Published:** 2022-05-24

**Authors:** Monazza Shahab, Samson Jamesdaniel

**Affiliations:** 1 Institute of Environmental Health Sciences, Wayne State University, Detroit, MI 48202, USA; fv9248@wayne.edu; 2 Department of Pharmacology, Wayne State University, Detroit, MI 48202, USA

**Keywords:** nitrative stress, auditory dysfunction, noise-induced hearing loss, ototoxicity, 3-nitrotyrosine, peroxynitrite

## Abstract

Nitrative stress is increasingly recognized as a critical mediator of apoptotic cell death in many pathological conditions. The accumulation of nitric oxide along with superoxide radicals leads to the generation of peroxynitrite that can eventually result in the nitration of susceptible proteins. Nitrotyrosine is widely used as a biomarker of nitrative stress and indicates oxidative damage to proteins. Ototoxic insults, such as exposure to noise and ototoxic drugs, enhance the generation of 3-nitrotyrosine in different cell types in the cochlea. Nitrated proteins can disrupt critical signaling pathways and eventually lead to apoptosis and loss of sensory receptor cells in the cochlea. Accumulating evidence shows that selective targeting of nitrative stress attenuates cellular damage. Anti-nitrative compounds, such as peroxynitrite decomposition catalysts and inducible nitric oxide synthase inhibitors, prevent nitrative stress-mediated auditory damage. However, the role of nitrative stress in acquired hearing loss and its potential significance as a promising interventional target is yet to be fully characterized. This review provides an overview of nitrative stress mechanisms, the induction of nitrative stress in the auditory tissue after ototoxic insults, and the therapeutic value of targeting nitrative stress for mitigating auditory dysfunction.

## 1. Introduction

Nitrative stress is a cellular stress phenomenon that is caused by the increased production and accumulation of reactive nitrogen species (RNS) intermediates and the inability of cells and tissues to remove these reactive products. Nitric oxide (NO), a key regulator of redox signaling, plays a central role in a number of physiological processes [1]. NO was first reported as a biological messenger molecule in 1987 when Bob Furchgott proposed that endothelium-derived relaxing factor (EDRF) might be NO [2]. The generation of NO is regulated by nitric oxide synthase (NOS) enzymes. Endogenously, three types of NOS exist, namely, the l-arginine-dependent neuronal nitric oxide synthase (nNOS), inducible nitric oxide synthase (iNOS), and endothelial nitric oxide synthase (eNOS) [1,2]. NO signaling is critical for vasodilation, bronchodilation, neurotransmission, inhibition of phagocyte and platelet aggregation, and inhibition of microbial activity [3,4,5,6,7]. The reaction of NO with partially reduced oxygen species or reactive oxygen species (ROS) can generate RNS intermediates such as peroxynitrite (OONO^−^), nitrogen dioxide (NO_2_), and dinitrogen trioxide (N_2_O_3_) [4]. NO and NO-derived RNS such as NO_2_ and N_2_O_3_ are implicated in neuronal cell death and apoptosis [3,4,5,6], while OONO^−^ causes lipid peroxidation and oxidation of protein-associated thiol groups [6,7]. Additionally, these RNS intermediates can induce posttranslational modifications, namely, 3-nitrotyrosination and nitrosylation, both of which have significant pathophysiological consequences [7]. The purpose of this review is to provide an overview of cochlear nitrative stress mechanisms with special emphasis on nitration and nitrosylation of cochlear proteins and their role in auditory dysfunction.

## 2. Nitrative Stress-Induced Post-Translational Modifications

**Figure 1:**
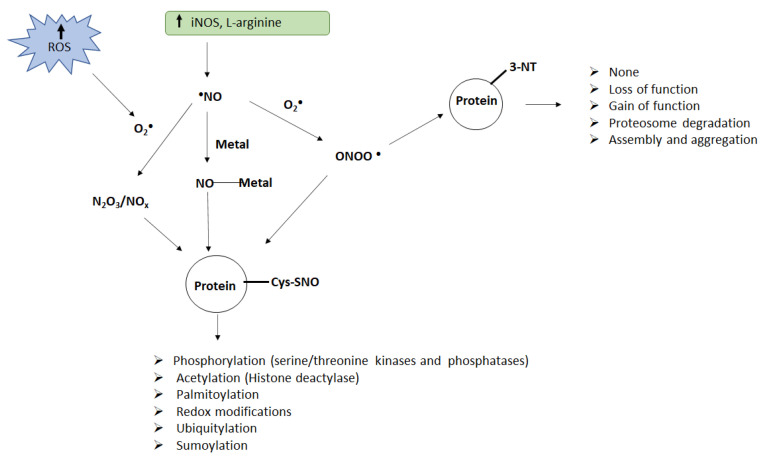
Schematic of protein nitration and nitrosylation and their consequences. Enhanced ROS generation and accumulation of nitric oxide can lead to the formation of peroxynitrite (ONOO−), NO oxides (N_2_O_3_/NOx), or metal–NO complexes (M–NO), which act as nitrating agents in vivo. Peroxynitrite can induce nitration of tyrosine (Tyr) residues on the proteins while peroxynitrite, metal–NO complexes, or NO oxides can induce nitrosylation of cysteine (Cys) residues on the proteins. Both nitration and nitrosylation can alter protein function resulting in several adverse consequences.

Protein nitration: Protein tyrosine nitration is a selective process where only a few proteins become nitrated on only one or few of the tyrosine residues [7,8]. Nitrotyrosine (3-NT) is formed by the substitution of a nitro group in place of hydrogen on carbon 3 in the phenolic ring of a tyrosine residue [8,9]. In vivo nitration mostly depends on superoxide (O_2_^−^) and NO as they react to form ONOO^−^. The accumulation of ONOO^−^ leads to the formation of peroxynitrous acid (ONOOH), which homolyzes to nitrite (NO_2_) and hydroxyl (OH) radicals or, in the presence of CO_2_, forms the nitroso-peroxocarboxylate adduct, which homolyzes to NO_2_ and CO_3_^−^ [9,10]. These one-electron oxidants (CO_3_^−^, OH, NO_2_) and Compound I of peroxidases can oxidize the phenolic ring of tyrosine to yield the tyrosyl radical (Tyr). The addition of NO_2_ to Tyr in a radical–radical termination reaction results in tyrosine nitration (Figure 1).

In addition, alternate pathways exist for nitration, especially those involving certain transition metal-containing proteins such as prostaglandin, endoperoxide H synthase, and MnSOD [8]. The analysis of different protein mixtures suggests that neither the abundance nor the number of tyrosine residues per protein molecules determine which protein is nitrated. However, some common features exist for proteins that are nitrated on tyrosine residues. These include (i) the presence of more acidic residues in the vicinity of target tyrosine (glutamic or aspartic residues), (ii) a small number of cysteine or methionine residues in the neighborhood of tyrosine residues, and (iii) the presence of loop- or turn-inducing amino acids such as proline or glycine. In basal conditions, the level of protein-bound 3-NT is low; however, in several pathological conditions, elevated levels of 3-NT are observed and used as a biomarker for these conditions [5,6,7,11,12,13,14].

Protein nitrosylation: S-Nitrosylation occurs by covalent attachment of a NO group to cysteine residue of specific proteins [15]. S-Nitrosylation of proteins can occur via (i) an oxidative pathway with NO in a higher oxidation status, (ii) a radical-mediated pathway with ·NO and thiol (RS·) radicals, (iii) metal-catalyzed RSNO formation in the presence of transition metals, and (iv) trans-nitrosylation [15] (Figure 1). The affinity of cysteine residues to NO can vary in different proteins. Although there are no general rules for determining which cysteine residues are susceptible to nitrosylation, analysis of NO transfer in proteins such as hemoglobin suggests the involvement of an acid–base motif in protein S-nitrosylation and denitrosylation [16,17]. The acid–base motif comprises flanking acidic (D, E) and basic (R, H, K) residues in the vicinity of reactive thiol cysteine sites. Hence, these can suppress or favor, respectively, the formation of nucleophilic thiolate through electrostatic interactions. Furthermore, this motif has been shown to be predictive in a number of cases. Additionally, the low pKa of cysteine also plays a role in S-nitrosylation [9]. It has been shown that the interaction between Cys thiols and aromatic side-chains in its vicinity promotes the formation of a thiolate anion, which enhances the potential for NO modification. S-Nitrosylated cysteines are also found in hydrophobic pockets of proteins [9], which can sequester or stabilize radicals to form S-nitrosylating species. To further understand and predict nitrosylated sites with accuracy computational studies are being employed. Unlike protein tyrosine nitration, protein S-nitrosylation is a less stable and easily reversible post-translational modification [18].

### 2.1. Pathophysiological Consequences of Nitrative Stress

Post-translational modifications determine the function, interaction, and signaling of proteins that eventually control the functional state of a cell. Both nitration and nitrosylation of proteins can interfere with signaling processes [15,19,20,21,22,23,24,25]. Although the nitration of tyrosine occurs at relatively low levels compared to tyrosine phosphorylation, it is more stable and capable of causing vital changes in biological function as it can modulate phosphorylation cascades. Furthermore, the addition of 3-NT can alter protein function because the binding of a nitro group on tyrosine reduces its pKa value from 10 to 7.2, which impacts its pH [9,26]. The development of pathological manifestations associated with protein nitration depends on the functional role of the specific proteins, whose nitration results in either loss or gain of function. Nitration of proteins such as MnSOD, glutathione reductase, prostacyclin synthase, and tyrosine hydroxylase leads to decreased activity, while cytochrome c, fibrinogen, glutathione S-transferase 1, and α-synuclein exhibit increased activity when nitrated [25,26,27,28,29,30,31]. Some proteins such as α1-chymotrypsin and transferrin do not show any change in function upon nitration [32]. Moreover, nitrated proteins are often targets for proteolytic degradation by the 20S proteosome. Although removal of modified proteins with altered function is essential, synthesis of new proteins is also critical for reestablishing homeostasis. However, if the rate of synthesis of a specific protein does not correspond with its rate of degradation, especially in proteins with long half-lives whose gene regulation may be less sensitive for changes, nitration can result in pathological phenotypes depending on the protein’s functional activity. Hence, establishing the identity of nitrated proteins is of paramount importance to envisage its functional implications.

S-Nitrosylation, another redox-based regulator of protein function, participates in a wide range of biological processes related to normal cellular function, as well as various pathophysiological conditions. Addition of S-nitrosothiols can affect protein activity such as translocation and protein function by modulating crosstalk between different molecules or by directly impacting the structure of the protein [16,18]. Furthermore, major protein post-translational modifications (acetylation, ubiquitination, sumoylation, etc.) are also affected and regulated by S-nitrosylation crosstalk. Protein S-nitrosylation can signal either cell death or cell survival according to the functional characteristics of its target protein. S-Nitrosylated proteins have been reported to regulate signaling pathways associated with neurodegeneration, apoptosis, cellular trafficking, DNA repair, muscle contractility, circulation, and cardioprotection [18,33,34,35,36]. S-Nitrosylation is easily reversible, which allows it to serve as an on/off switch to precisely modify protein function in response to cellular signals. Denitrosylation occurs through enzyme-mediated reactions or nonenzymatically by changes in the redox environment of the protein. Depending on the levels of cellular oxidative stress, this reversible S-nitrosylation, which plays a crucial role in NO cell signaling, can progress to an irreversible sulfonic acid modification resulting in cellular toxicity [18]. Protein S-nitrosylation plays a central role in regulating stress-induced apoptosis as it can signal either a pro- or an antiapoptotic response, according to the characteristics of its substrate protein [4]. Such stimulatory and inhibitory signaling allows S-nitrosylation to regulate both mitochondrial and nuclear programs of apoptosis and fine-tune the cellular responses to apoptotic stimuli [5,33]. 

### 2.2. Nitrative Stress and Otopathology

Nitrative stress has been reported in different types of auditory dysfunction such as noise-induced, age-related, and drug-induced hearing loss [37,38,39,40,41,42,43,44,45]. Although both protein nitration and S-nitrosylation can lead to cellular apoptosis, there are very few studies that reported the association of S-nitrosylation with cell death in the auditory system. The induction of nitrative stress is determined by measuring the changes in related biomarkers in the inner ear. Several studies have reported the association of nitrative stress with auditory dysfunction; however, very few studies have conducted an in-depth investigation of the associated signaling mechanism. This section briefly describes the otopathological conditions in which nitrative stress plays a role in causing auditory dysfunction. A list of studies that detected nitrative stress in the inner ear and/or analyzed their association with auditory dysfunction is provided in Table 1.

The link between noise-induced hearing loss (NIHL) and nitrative stress is well documented [14,21,22,23,45,46]. Studies on rodents (BALB/c mice or CBA/J mice or Wistar rats) indicated that ototoxic insults with either noise or ototoxic reagents increased the generation of RNS, and pretreatment with anti-nitrative agents protected against cochlear injury [43,47,48,49]. Both ROS and RNS were implicated in the loss of outer hair cells (OHCs) after noise exposure [23,39,44,50]. NO accumulation in the mitochondria of OHC during NIHL was reported to interfere with mitochondrial function in the guinea pig’s cochlea [41]. Noise exposure was also associated with an increase in the expression of endothelial nitric oxide synthase (eNOS) and induction of inducible nitric oxide synthase (iNOS) in the cochlea [50]. Furthermore, noise exposure increased the levels of 3-NT, a biomarker of nitrative stress, in the lateral wall, modiolar region, OHC, inner hair cells (IHC), and pillar cells in the guinea pig cochlea [22,43,47,48]. Studies on the relationship between autophagy and oxidative stress in NIHL indicated that 3-NT was elevated in OHC [23]. The accumulation of ROS/RNS after noise exposure activated the intrinsic Caspase-mediated apoptotic pathway via JNK and p38 MAPK signaling, resulting in cochlear cell death [43].

Nitrative stress also plays an important role in drug-induced hearing loss, especially after treatment with anti-cancer drugs such as cisplatin [47,48,49] or aminoglycosides such as gentamicin [51]. Anticancer drug cisplatin damaged the inner ear through generation of ROS and RNS and via the formation of DNA adducts [47]. The cisplatin-induced increase in the levels of 3-NT and peroxynitrite generation in mice cochlea accompanied the onset of cochlear apoptosis and hearing loss [47,48]. In addition, the levels of iNOS activity and Nf-κB were found to be significantly elevated. A strong correlation was observed between cisplatin-induced hearing loss and nitrative modifications of cochlear proteins in a dose-dependent manner. The most abundantly nitrated protein in the cochlea after cisplatin treatment was LMO4, a transcription factor regulator [47]. Nitration of Tyr-65 and Tyr-77 was detected in LMO4 by mass spectrometry. LMO4 levels were also decreased with cisplatin treatment, suggesting that this could be due to the degradation of nitrated LMO4, one of the consequences of protein nitration. In vitro studies with UB/OC-1 cell lines (derived from embryonic mouse inner ear) also verified the role of nitrative stress in cisplatin-induced ototoxicity [48,52]. These studies indicated that a marked increase in cell death, as well as Caspase-3 expression, a marker of apoptosis, after cisplatin treatment correlated with the increase in 3-NT levels [47]. This association was also detected in nonmammalian models such as zebrafish, where cisplatin-induced loss of hair cells in the neuromasts was accompanied by elevated expressions of 3-NT, decreased LMO4, and increased apoptosis [53]. Similarly, treatment of mouse organotypic cultures with gentamicin increased the 3-NT levels in the OHCs, indicating the induction of nitrative stress in aminoglycoside-mediated ototoxicity [51].

Nitrative stress was also reported in other otopathological conditions. Immunoreactivity to 3-NT was elevated in the Deiters’ cells, pillar cells, spiral ganglion cells, stria vascularis, and spiral ligament of aged CBA/J mice [54]. Immunostaining of 3-NT was found in vascular endothelial cells of the blood–labrynthine barrier in Meniere’s disease patients [55]. Together, these studies suggested that nitrative stress is a key factor in many otopathological conditions.

### 2.3. Assessment of Nitrative Stress

The effectiveness of interventional strategies that attenuate stress-induced damage to cells often depends on the early detection of oxidative/nitrative stress. Several molecules have been identified as reliable biomarkers of nitrative stress. Detection of these biomarkers is not only valuable for diagnostic purposes but also useful for assessing prognosis and determining the therapeutic value of interventions. A number of techniques are employed to detect different markers of nitrative stress in in vivo models. However, it is challenging to employ many of them for assessing the damage in the auditory organ because of the difficulty in accessing the inner ear and the short half-life of RNS such as ONOO^−^. For example, electrochemical sensors are used in other models to detect ONOO^−^ released from living cells in trace amounts but are yet to be employed in the auditory system. Nevertheless, selective NO electrodes and electrometers have been used to assess NO concentration in the perilymph [44]. Changes in NO levels in auditory dysfunction can also be assessed using fluorescent dyes such as 4,5-diaminofluorescein diacetate (DAF) and 4,5-diaminofluorescein diacetate-2 (DAF-2), which probe for NO production in cells [44]. In addition, a chemiluminescence assay using nitrate reductase can be used to detect NO in the inner ear. The NOS family of enzymes that enable the production of NO are assessed as an indirect measure of nitrative stress. The expression of iNOS in the auditory organ has been reported in many studies and is measured by employing immunohistochemistry and/or immunoblotting methods [50]. However, the detection of 3-NT in cochlea appears to be the most commonly employed approach for measuring nitrative stress in the auditory system. Techniques such as immunoblotting, immunocytochemistry, and mass spectrometry are employed to detect 3-NT in the inner ear and to identify nitrated cochlear proteins [47,48,49]. Together, these biochemical techniques have enabled us to gain important insights into the role of nitrative stress in auditory dysfunction.

### 2.4. Targeting Nitrative Stress for Otoprotection

Numerous studies have indicated that nitration of proteins can be reversed, and targeting nitrative stress is a promising interventional strategy for mitigating oxidative damage to the cochlea [48,52,56,57,58]. Protein nitration and its signaling have been targeted to prevent apoptosis in organs such as the eye, pancreas, and kidney [59,60]. Unlike ROS, which has a role in normal cell signaling, a beneficial role of ONOO^−^, which causes cochlear damage, has not yet been reported in the auditory system. Although nitration of proteins occurs in tissues under physiological conditions [61], the use of peroxynitrite decomposition catalysts (PNDCs) does not interfere with physiological functions and, to date, no evidence exists that selective targeting of peroxynitrite has any side-effects. Treatment of mice with PNDCs such as SRI110, a metalloporphyrin, attenuated the cisplatin-induced increase in the levels of 3-NT and Caspase-3, while auditory brainstem responses (ABR) of 6 week old CBA/J mice indicated that SRI110 cotreatment mitigated cisplatin-induced hearing loss [48]. Further investigation of the role of PNDCs in mitigating cisplatin-induced ototoxicity suggested that SRI110 cotreatment inhibited cisplatin-induced protein nitration, prevented cisplatin-induced inactivation of Stat3 protein, a critical protein required for activating the Jak1/Stat3 pathway, and enhanced antiapoptotic signaling in UBOC1 cell lines [57]. Compounds such as ebselen, which is both a glutathione peroxidase mimic and an excellent scavenger of peroxynitrite radicals, and antioxidants, such as Trolox, were used to prevent the nitration of tyrosine in cochlear proteins and protect the cochlea from cisplatin-induced damage and hearing loss [47,62,63,64]. Although a direct link between inhibition of nitrative stress with ebselen and attenuation of NIHL is yet to be reported, phase 2 clinical trials in healthy adults aged 18–31 years indicated that 400 mg of ebselen treatment twice daily prevented temporary and permanent noise-induced hearing loss [64]. Treatment of mice with antagonists of nitric oxide synthase such as L-NAME attenuated the noise-induced increase in the level of 3-NT [43,58]. Additionally, compounds such as sodium butyrate, adenosine amine congener (ADAC), polyphenols, Astragaloside IV, L-cysteine, and N-acetyl cysteine (NAC) were reported to prevent cochlear nitrative stress and hearing loss [56,65,66,67,68,69]. Recent findings from our laboratory showed that selective scavenging of ONOO^−^ using pure MnTBAP prevents the cisplatin-induced decrease in the amplitude and increase in the latency of ABR wave I in CBA/J mice, which suggests that targeting nitrative stress also prevents synaptic dysfunction and hidden hearing loss (unpublished data). Thus, compounds that target nitrative stress are increasingly used to confer significant otoprotection after noise- or drug-induced cochlear damage and hearing loss. More importantly, unlike antioxidants that were used to target oxidative stress for mitigating ototoxicity induced by the anticancer drug cisplatin, selective inhibition of nitrative stress by PNDCs does not interfere with the anticancer activity of a drug. Therefore, targeting nitrative stress meets the critical criteria for an effective therapeutic (i.e., otoprotective, safe, and well-tolerated, without compromising the activity of the primary drug). A list of studies that employed different pharmacological compounds to inhibit nitrative stress in the inner ear is provided in Table 2.

## 3. Conclusions

Overall, a plethora of studies have indicated that nitrative stress is an important contributor to auditory dysfunction after exposure to noise or other ototoxic agents. Although the number of studies on the role of S-nitrosylation in auditory dysfunction is limited, recent technological advancements have enabled the detection of multiple biomarkers of nitrative stress in the auditory organ, identification of nitrated cochlear proteins, and delineation of associated signaling mechanisms. Compelling evidence from recent studies indicated the otoprotective efficacy of anti-nitrative compounds. However, this promising target is yet to be fully exploited for treating sensory neural hearing loss. Moreover, the potential role of nitrative stress in sensorineural hearing loss induced by viruses and autoimmune disorders is yet to be fully understood [70,71]. Continued progress in understanding the role of protein nitration and nitrosylation mechanisms in different otopathological conditions would enable the design of effective interventional strategies and development of a potential class of novel drugs for acquired hearing loss.

## Figures and Tables

**Table 1:** Studies showing nitrative stress in auditory dysfunction.

Author/Year	Model (Animal/Cell Culture)	Nitrative Stress Molecule	Ototraumatic Agent	Region Affected in Inner Ear
Alvarado et al., 2015	Wistar rats	nNOS	Noise	Spiral ganglion, spiral ligament, and cochlear nerves
Han et al., 2013	Guinea pigs	3-NT	Noise	OHCs, IHCs, spiral ganglion, and pillar cells
Jamesdaniel et al., 2012	UBOC1 cell lines	3-NT	Cisplatin	UBOC1 cells
Jamesdaniel, 2014	Wistar rats	3-NT	Cisplatin	Spiral ganglion, stria vascularis, and organ of Corti
Jamesdaniel et al., 2016	UBOC1 cell lines	3-NT	Cisplatin	UBOC1 cells
Jiang et al., 2007	CBA/J mice	3-NT, OONO^−^	Aging	Deiters’ cells, pillar cells, stria vascularis, spiral ganglion
Lu et al., 2011	Rats	3-NT	OONO-	Spiral ganglion neurons
Lynch et al., 2005	Female rats F-344	3-NT	Cisplatin	OHCs
Nagashima et al., 2010	Std-ddY mice	3-NT	Noise	Spiral ligament
Pourbakht et al., 2005	Guinea pigs	3-NT	Noise	OHCs
Rathinam et al., 2015	UBOC1, HK-2, and SH-SY5Y cell lines	3-NT	Cisplatin	UBOC1, HK-2, and SH-SY5Y cells
Rosati et al., 2019	UBOC1 cell lines	3-NT	Cisplatin	UBOC1 cells
Shahab et al., 2020	Zebrafish	3-NT	Cisplatin	Hair cells in neuromast
Shi et al., 2002	Guinea pigs	3-NT	Noise	OHCs
Vlajkovic et al., 2010	Wistar rats	3-NT	Noise	Organ of Corti, spiral ganglion neurons, spiral ligament, spiral limbus, inner sulcus cells, inner phalangeal cells, pillar cells, Deiters’ cells, Hensen’s cells
Wu et al., 2020	CBA/J mice and HEI-OC1 cells	3-NT	Noise	OHCs
Xiong et al., 2011	Guinea pigs	3-NT and iNOS synthase	Noise	Stria vascularis, spiral ligament, organ of Corti
Yamasoba et al., 2007	Guinea pigs	NO	Noise	Organ of Corti, afferent dendrites beneath IHCs
Yamashita et al., 2004	Guinea pigs	3-NT	Noise	Spiral ganglion, organ of Corti, lateral wall
Yamashita et al., 2005	Guinea pigs	3-NT	Noise	OHCs
Yang et al., 2017	Guinea pigs	3-NT	Noise	OHCs
Yuan et al., 2015	CBA/J mice	3-NT	Noise	IHCs and OHCs

**Table 2:** Studies showing inhibition of nitration and attenuated hearing loss and auditory dysfunction.

Author/Year	Model (Animal/Cell Culture)	Inhibitors of Nitration	Biochemical Effect of Intervention	Outcome of Intervention
Diao et al., 2007	Guinea pigs	NG-Nitro-L-arginine methyl ester (L-NAME)	Decreased NO production	L-NAME protected the cochlea after noise exposure
Jamesdaniel et al., 2012	Wistar rats	Trolox	Decreased nitration of cochlear proteins	Attenuated cisplatin-induced OHC loss and hearing threshold shifts
Jamesdaniel, 2014	Wistar rats	Trolox	Decreased nitration of cochlear proteins	Attenuated cisplatin-induced ototoxicity and nitration of cochlear proteins
Jamesdaniel et al., 2016	UBOC1 cell lines	SRI110	Decreased 3-NT	SRI110 inhibited cisplatin-induced cytotoxicity
Jia et al., 2018	HEI-OC1 cells and explanted cochlear tissue	Tauroursodeoxycholic acid (TUDCA)	Decreased NO production	SRI110 inhibited cisplatin-induced cytotoxicity
Lu et al., 2011	Rats	L-Cysteine	Decreased peroxynitrite	Decreased gentamicin-induced ototoxicity
Lyncha et al., 2005	Fisher-344 rats	Ebselen	Decreased lipid peroxidation	Protected cochlea from cisplatin-induced OHC loss and hearing loss
Nagashima et al., 2010	Std-ddY mice	Tempol and Nω-nitro-L-arginine methyl ester	Decreased 3-NT and 4-HNE	Protected noise exposed animals from hearing loss
Pourbakht et al., 2005	Guinea pigs	Ebselen	Decreased peroxynitrite	Reduced the noise-induced permanent threshold shifts
Rosati et al., 2019	UBOC1 cell lines	SRI110	Decreased 3-NT	Prevented cisplatin-induced cytotoxicity
Sánchez-Rodríguez et al., 2016	Sprague-Dawley Rats	Polyphenols	Decreased 3-NT and superoxide anions, increased glutathione peroxidase and SOD	Protected against age-related hearing loss
Wu et al., 2020	CBA/J mice and HEI-OC1 cells	N-Acetyl cysteine (NAC)	Decreased 3-NT and superoxides	Prevented loss of OHCs induced by noise exposure
Vlajkovic et al., 2010	Wistar rats	Adenosine amine congener (ADAC)	Decreased 3-NT	Attenuated hearing threshold shifts after noise exposure
Xiong et al., 2011	Guinea pigs	Astragaloside IV	Inhibited iNOS	Protected the cochlea from noise-induced damage
Yamashita et al., 2005	Guinea pigs	Salicylate and trolox	Decreased 3-NT and superoxides	Reduced ABR shifts post noise exposure
Yamasoba et al., 2005	Guinea pigs	Ebselen	Decreased NO production	Reduced ABR shifts post noise exposure
Yang et al., 2017	Guinea pigs	Sodium butyrate (SB)	Decreased 3-NT and HDAC1	Attenuated noise-induced loss of OHC and hearing loss

## Data Availability

Not applicable.
